# Harnessing mobile devices to support the delivery of community-based clinical care: a participatory evaluation

**DOI:** 10.1186/s12911-019-0869-x

**Published:** 2019-07-16

**Authors:** Jasmine Harvey, John Powell

**Affiliations:** 1 Nuffield Department of Primary Care Health Sciences, University of Oxford, Radcliffe Observatory Quarter, Woodstock Road, Oxford, OX2 6GG England; 2 Division of Primary Care, School of Medicine, University of Nottingham, Tower Building, NG7 2RD Nottingham, England

**Keywords:** Mobile health systems, Apple iPads, Implementation, Adoption, Participatory approach, Focus groups, Co-construction, Nurse technology fund, Digital health, NHS, Mobile working

## Abstract

**Background:**

A large provider of community health services (an NHS Trust in England) deployed Apple iPads to its front-line community-based healthcare clinicians (predominantly nurses) to enable them to increase responsiveness to patients’ and their families’ needs. We conducted a participatory formative evaluation of this iPad initiative among different users and the informatics teams implementing it, to establish how such initiatives can sustain adoption and achieve their stated benefits.

**Methods:**

We used a participatory approach involving a partnership between study investigators and key decision-makers of the initiative to engage stakeholders in the study. Methods included focus groups and group discussion, meetings with key personnel and analysis of documents related to the initiative. Using a participatory technique, members of the organisation identified practical challenges to inform the on-going process of implementation and adoption in the Trust.

**Results:**

Healthcare professionals identified many benefits associated with having iPads to support care delivery, including streamlined workflows and accessible information at the point-of-care in the community. However, challenges that interfered with implementation were also reported by both the team implementing the initiative (IT team) and early users. Challenges reported by IT team are: adopter clinicians’ scepticism and suspicion; clinician non-compliance with training and operational guidance procedures; and managing adopter expectations. Challenges reported by users are: setting-up and maintaining the devices on a long-term basis; blurring of personal and professional boundaries; and disconnection from the IT team. Results show that these challenges could be overcome if there were more informal ‘socialised’ interactions between adopters and between adopters and the IT team.

**Conclusions:**

We suggest that similar initiatives require increased ongoing dialogue between different levels of stakeholder groups, in the form of socialised engagements, to avoid common misunderstandings and to promote the processes involved in co-constructing the initiative on a generally-agreed and sustainable basis.

**Electronic supplementary material:**

The online version of this article (10.1186/s12911-019-0869-x) contains supplementary material, which is available to authorized users.

## Background

Health providers are increasingly harnessing mobile computing tools to support the delivery of care remote from the hospital site. Tablet devices are increasingly becoming part of the enabling tools used by nurses and other healthcare professions because of their potential to save time, increase access to information and facilitation of timely and accurate documentation. However, research regarding the impact of technology on nurses and their work is limited [1]. In England, a large provider of community health services (an NHS Trust) deployed Apple iPads to its front-line community-based healthcare clinicians (predominantly nurses) to enable them to increase responsiveness to patients’ and their families’ needs. We use the labels 'clinicians', 'community nurses' and 'nurses' interchangeably in this article.

Telemedicine is defined broadly by the World Health Organization as the delivery of health care services at a distance using electronic means for “the diagnosis of treatment, and prevention of disease and injuries, research and evaluation, education of health care providers” [2, 3]. While the iPad initiative was not primarily considered a telemedicine solution, as community nurses used them to store and transmit notes from in-person consultations, the devices were able to support telemedicine as clinicians in remote places within the community were able to share information (e.g photos of wounds) to confirm diagnosis or treatment.

In this paper, we identify the lessons learned from this initiative (referred to as the *iPad initiative* in this document) and draw conclusions for future research and practice.

After an initial pilot phase to identify an acceptable and user-friendly device, approximately 1,000 Apple iPads were deployed within the organisation to front-line community-based healthcare clinicians. Subsequently, further funding was obtained, and an additional 2,000 iPads were deployed over a three-month period to community psychiatric nurses, district nurses, psychologists and health visitors.

The iPads have built-in security which includes the ability to track and locate the device if lost or stolen. For an additional level of management and security purposes, the iPads were managed using a Mobile Device Management (MDM) solution. MDMs have a significant number of features that address concerns related to data privacy, security, segregation of personal / private data, and so on. The list of features available differ by MDM supplier and in this case, all iPads were managed by a custom in-house security system (details unspecified for security reasons). All of the iPads were provisioned with Wi-Fi and a 3G/4G mobile data connection. Initially, each iPad had a specified amount of mobile data available for use before additional charges were applied. This was subsequently replaced with an organisation-wide contract for mobile data, whereby one combined monthly data bundle was shared across all devices. Whilst individual user’s mobile data usage was monitored, this was done in the context of educating the user to alternatives when mobile data usage was identified as excessive. One example of how users sought alternatives was asking patients to allow them to use Wi-Fi whilst in a patient’s home.

Community nurses had to ask patients for consent to use their iPads for storing notes, asking patients to allow them to use Wi-Fi became part of the routine of obtaining consent to securely transmitting patient notes using the Carenotes App. Of note, participants of this study did not report refusal of consent by any patient in relation to using the CareNotes system to transmit their information".

The devices were configured with local (Trust) resources such as email, calendar, other browser-based services and professional-based applications (apps). The latter included the Patient Case Management Information System (PC-MIS) used by the Improving Access to Psychological Therapies (IAPT) services, an online-based programme that supports trainees, trainers and service teams offering interventions aimed at treating people with depression and anxiety disorders [4]; a self-management tool for monitoring the moods of people with bipolar disorder; and a collection of patient-facing mental health apps available in the NHS choices health apps library (apps.nhs.uk).

A key purpose of the initiative was to make the mobile version of the Trust’s new Electronic Health Records (EHR) system, called Carenotes, available. It was expected that the devices would enable clinicians to perform various tasks including: access department-specific clinical information records from anywhere, read and write offline assessments and clinical notes at the point of care directly into the EHR, conduct clinician to clinician and clinician to patient video conferencing using FaceTime/Skype apps, and negate the need for time-consuming journeys, enable staff to use various apps to collect patient outcome scores, and provide patients with real-time interactive teaching and guidance thereby improving the clinical encounter. The iPads also provided access to general resources for both professional and personal use. The anticipated benefits of the iPad initiative were: productivity gains in terms of reducing time taken to perform tasks, cost savings through facilitating mobile offices and enabling the downscaling of costs associated with traditional offices, improvements in real-time clinical decision-making and reduction of life-threatening errors.

The aim of the study was to identify critical processes in the implementation process that could help sustainable adoption.

## Methods

### Theoretical underpinning

There are several theoretical approaches to developing knowledge about an on-going implementation process [5]. We adopted a rarely used approach in healthcare adoption studies, the participatory approach, which has some similarities with the stakeholder approach. In the participatory approach, methods are qualitative techniques that holistically engages small section of the user population. Whereas the participatory approach engages key stakeholders’ insider knowledge of the implementation process, the stakeholder approach to evaluation, however, tends to be limited to eliciting and summarising the opinions of stakeholders in a project [6–8]. To fully engage stakeholders in the processes of evaluation, we used a participatory approach. Cousins and colleagues define participatory evaluation as applied social research that involves a partnership between trained evaluation personnel and practice-based decision makers, organisation members with programme responsibility, or people with a vital interest in the programme that is being evaluated [6, 7]. Three key ways in which the participatory evaluation model differs from other evaluation models are firstly that it tends to focus on a small number of key users. Secondly that these users are not merely consulted but participate collaboratively in formulating the problem, designing the methods, and analysing and interpreting the findings. Thirdly that while the evaluator coordinates the project, the users are jointly responsible for the study.

Key features of the participatory approach described by Cousins and Earl [7] and Robinson et al. [6] were used to guide how data was collected and analysed. Participatory evaluation methods can include the following: meetings, interviews, focus groups, diagrams, images and video [8, 9].

### Data collection

The following methods were employed:

- Meetings with management staff leading the initiative and its delivery, to understand the nuts and bolts of the initiative before data collection commenced.
- Secondary documentation associated with the iPad initiative was analysed. Documentation included in the Trust’s original proposal for the initiative, the funding application and a benefit realisation plan.
- Three focus group sessions were conducted with primary users of iPads from three directorates in the Trust. The directorates were the young people’s directorate, the adult directorate and the older adult directorate. Participants were chiefly community nurses who travel into patients’ homes to delivery care (see Additional file 1 for focus group template)
- One group discussion session with key members of the team administering the iPads (henceforth referred to as the IT team) was conducted to discuss experiences, lessons learned, and challenges regarding implementation.
- A collaborative document was created to capture synergies between intended benefits of the iPad initiative and evaluation goals. This process was ongoing throughout the evaluation.

#### Participants recruitment for group sessions

In line with the participatory approach, members of the IT team were involved in recruiting participants to the focus group sessions. Participants were initially identified from the IT team’s database of primary users. Primary users were front-line clinicians who conducted community visits with patients. Even though participants were identified by the IT team, they had to consent to be part of the study. Not all participants identified consented. Once a participant initially agreed to take part in the study, their details were forwarded to the lead evaluator of the iPad initiative (JH). JH then formally recruited the participant to the study by sending out a participant information sheet (PIS) detailing purpose of study, and later obtaining written consent. Through this process, 20 participants were recruited for the group sessions:

- five participants from the adult directorate,
- six participants from the older adult directorate,
- three participants from the young people’s directorate (including one joining the session remotely via FaceTime) and
- six participants from the team implementing the iPad initiative (IT team).

The group sessions were conducted in December 2015 and took one hour on average. Although participants were not given incentives to take part in the study, food and drinks were provided during the sessions. The sessions were audio-recorded and transcribed verbatim (averaging 13,000 words per transcript). Transcripts were anonymised and analysed. Each session was moderated by JH, who took notes in addition to the audio-recording.

### Analytical framework and data analysis

Participatory analysis, combined with socio-technical approaches, was used to inform the analytical framework. For example, while constructivist approaches of co-construction were used to analyse the text [10], participatory analysis techniques such as involving stakeholders in the analysis and findings were used to finalise results [7, 8]. To involve stakeholders in the analysis and interpretation of findings, JH engaged with the Director of Informatics at the NHS Trust by sharing outcome summaries from each focus group immediately after each session. These summaries (initial findings) were also shared with two project managers who were involved with scoping the evaluation, and with each focus group participant. In addition, JH shared a compilation of findings from the deeper analysis with the Director of Informatics who, reciprocally, shared some survey results from users of the iPad to complement our findings.

Data from the group sessions were coded in Quirkos software version 1.3 (https://www.quirkos.com/) using the categorisation method for qualitative analysis. All categories were printed out and excerpts were analysed (and recoded if necessary) into themes. Themes were further analysed to explore research questions relating to the stated benefits of the iPad initiative and to the co-construction of technology for sustainable adoption through learning from both implementers and adopters. For example, we considered implementer experiences of certain issues and how users related to these issues in their experiences, and vice versa. The themes of the results are discussed from both implementer and user perspectives in the following section (3) in terms of practical challenges and suggestions for a sustainable initiative.

## Results

### Practical challenges for implementers

Although the initiative had been active for just a year at the time of the study (2015), the IT team reported getting satisfaction from seeing opinions change from negative to positive as users adapted to their iPads. The IT team also reported realising benefits early on, in the form of users reporting that the iPad was facilitating mobile work and reducing requirements for extensive Trust resources.

However, while discussing key points in their delivery strategies, the IT team identified some practical challenges that they had faced, and lessons learned from the challenges. Three key challenges discussed were *adopter clinicians’ scepticism and suspicion*, *clinician non-compliance with training and operational guidance procedures*, and *managing adopter expectation*.

#### Challenge one: user scepticism and suspicion

The team admitted that while the strategy for distributing the iPads “*was pretty much first come first serve, it was advertised to all community staff*”, there was a clear goal to get front-line community nurses using the devices as soon as possible. Hence, the objective was to mobilise managers within the directorates to select people in their respective teams to receive iPads: “*The main strategy was just to put iPads into clinician’s hands [erm] and if we got rid of all of them, great and if we didn’t then we needed to kind of rethink.” However,* the IT team found distribution of the devices challenging, because staff did not know why they were suddenly getting free iPads and became suspicious. To the team, the general thought among clinicians was that the Trust had to have a hidden agenda if free iPads were being given away, as succinctly expressed by this interviewee:

*“What we found [erm] was that people were kind of sceptical to begin with about coming to get an iPad because they thought there was some kind of hidden agenda and that we would ask them to do something in return.” (Participant 5, IT team)*

The IT team reflected that when faced with scepticism and suspicion, the most effective way that they resolved these issues was to reemphasise benefits to users, which involved reiterating statements along the following lines: the structure of the clinician’s day, as understood by the IT team, is back and forth to base, or to different appointments, and then perhaps go back to base at the end of appointments to type up notes. The new system would be able to change the structure of the clinician’s day so that they can do all their visits and then maybe finish off for an hour at home, or finish off in the office at the end of the day, ultimately improving efficiency and reducing travel time.

#### Challenge two: clinicians’ misunderstanding of training procedures

Two types of tutorial sessions, a long and a short session, were conducted over a three-month period to train clinicians before using the device. Clinicians were asked to make certain preparations before attending. A key challenge faced in the longer tutorials was that some clinicians did not make those required set-up preparations before arriving. However, this was resolved by employing IT team members whose task was to help those lagging behind in mini walk-arounds. The challenge in the shorter drop-in sessions was that it appeared that clinicians had misinterpreted what a drop-in meant, or did not have the right information. Clinicians, it appeared, expected to drop-in any time of the day to pick up an iPad and do a quick tutorial rather than attend at specific drop-in times. The following extract highlights these challenges.

*“We asked them to do certain set ups before they arrive, which was to get their Apple ID set up. Quite a few people didn't. Had a sort of mini session while the main session was going on where we were helping those people get up to speed.” (Participant 2, IT team)*

Lessons learned therefore were to provide clearer communication to clinicians about when to attend training and what it involves; and getting clinicians to understand this and act accordingly.

#### Challenge three: managing user expectations of device functionality and apps

An objective of the early deployment of iPads to clinicians was for them to become familiar with the device to enable easy adaptation to the Carenotes iOS app later. Clinicians had the responsibility to download the latest apps and upgrades onto their respective devices for maintenance. Although the IT team learned that users adapted to different functions on the device depending on what was most useful for their needs, a key challenge was that some users did not know which functions the apps performed, or what they could get out of devices. This, as discussed by the IT team, became apparent when the Carenotes iOS app was deployed. The team reflected that, as a result, managing expectations became a key factor in the implementation.

Strategies to manage expectations involved pushing device updates to users, conducting mini visits to discuss challenges and organising more tutorial sessions. However, even after these ‘managing expectation’ strategies were in place, some clinicians were still not comfortable maintaining their devices independently – especially, as shown by this excerpt, if they had to download clinical apps to update the devices:

*“There was just a few people that were a bit worried about updating it, because of what they’ve got on there. … But you're always gonna get those people that will need kind of the hand hold.” (Participant 1, IT team)*

### Practical challenges for users

User participants discussed many benefits of the iPad, especially for mobile or remote work, which according to one, “*improves the quality of care we can provide*. *It’s improved quality of our work experience … I know it’s expensive, but it’s not a massive cost when you consider the benefits that we are getting out of it.*” Benefits participants discussed included: the iPad being paperless, thus enabling them to store electronic versions of paper documents; better organisation of work routines and practices for a smoother workflow; increased interactivity with patients during visits and therapeutic sessions for collaborative treatment; and the iPad enabling real-time consultation with colleagues. All of these gains have helped the Trust achieve its objectives for deploying the initiative.

In general, user attitudes towards the iPad initiative were positive because it had changed their work practices for the better and, as such, 100% said they would recommend the device to colleagues and would prefer the initiative to continue:

*“I didn't have an iPad until I had this. And now if they try to take this iPad off me it would hurt.” (Female, older adult directorate)*

Although participants were satisfied with their devices and professed that they ‘now’ could not do without them, they also identified some challenges for adoption. A range of challenges were identified: some complained about having to buy apps that should be available for free, and some thought that losing connection in rural locations was a threat to long-term use of the iPads, as well as having a data usage limit on the devices. As the excerpts below show, participants who were not aware of the limit on data usage changed their usage habits when they became aware of it.

*“We got an email recently to say some people had been going over their limits and can people be aware of when they are using it and use it as much as you can on Wi-Fi and not on the 4G or 3G. When I started I was never told. All I was told was, you can use it for personal use as well and I went, eh. I know people do take them away on holiday to use, but I wasn't aware there was a limit. I hadn't been told that I'd gone over mine, so I am presuming I haven't” (Source 2).*

*“I hadn't heard of anyone being charged” (Source 3 Female); “I must admit that. It's not a problem of mine. I've always been working on the principle that the Trust have a good deal for allowing 3G” (Source 3 Male).*

The biggest complaints, however, concerned *setting-up and maintaining the devices on a long-term basis*, *blurring of personal and professional boundaries*, and *disconnection from the IT team*, all of which are discussed in the following sub-headings.

#### Challenge one: the work of maintaining and updating the device

This challenge is the antithesis of, but also parallels, the IT team’s third challenge, ‘managing user expectations of device functionality and apps’. While some users felt challenged by the work required to keep the devices updated and maintained, others were simply overwhelmed by the tasks involved, as this extract shows:

*“I got an email saying, right, you have to do these 29 steps and then you let us know and then you have to do these 30 steps. It was just like, I mean, probably weren't that big … But I just went, do you know what, I haven't got time. And so I've never [downloaded] the Carenotes app again, because I just thought, I can't do that. So if somebody had come out and said, well, we'll come and do it, we'll sit and log in, that would have been fine.” (Female 1, adult directorate)*

#### Challenge two: blurring of personal and professional lives

Another key challenge which user participants discussed concerned privacy issues surrounding use of the iPads and how the device has contributed to the blurring of their professional and private lives. At the time of the study, clinicians were allowed to use the devices for personal as well as professional purposes. This appeared to be a benefit in many cases and had increased adoption and use of the devices. However, it also seemed to be preventing some from fully utilising features on the devices because of privacy concerns, as these excerpts demonstrate:

*“I suppose the negative aspect of it is that, actually the kind of line between your personal life and your work life is starting to blur.” (Male, young people’s directorate)*

*“It is a bit more of a grey—I've got my own emails coming through on this as well. So sometimes they come through and you are like, uh. It works both ways. Sometimes often on a Sunday night looking at your work emails and then you get—it's been one of them grey areas.” (Female, older adult directorate)*

#### Challenge three: disconnected communication channels to IT team

User participants were largely unhappy with the level of information they were receiving from the IT team, and the channels through which they were receiving the information. Users reported at the time of study that information on updating new devices, new apps and educational material was not located in a central space for convenient access, but rather dispersed through channels such as emails and pop-up notes on the screen. Users found the dispersed channels of information unhelpful and sometimes distracting, especially if notes popped up while they were trying to complete clinical tasks. They therefore suggested a central source of information and a clearer IT enquiries system, a regular newsletter on updates, notices and new applications, and iPad champions embedded into user teams to increase communication and interactivity.

Key challenges the implementer, and adopters identified are listed in Table 1.

**Table 1:** A summary of key challenges identified from implementer and user perspectives

Implementer (IT Team) key challenges. *Taken from 6 key member from the IT team*	End user (community nurses) challenges *Taken from 20 early adopters of the CareNotes service*
Clinicians (adopters) were suspicious that iPads were being given out for free and wondered what information they were stealthily giving in return for using the devices.	Community nurses found that processes involved in updating and maintaining the devices needed too much effort.
The IT team required more effort than they anticipated to train nurses to use devices effectively. Some nurses did not take part of training or came into training sessions unprepared.	Community nurses found themselves using iPads for work purposes when at home.
Effort was needed by the IT team to manage nurses' expectation that the devices will solve all mobile work issues at immediately.	Nurses were concerned that it was not clear who they should contact if they had a problem with their devices.

## Discussion

For a technological service to be co-constructed and effectively translated into practice, many critical factors have been identified in the literature. Factors such as simplicity, ease-of-use, and compatibility with existing values and practices [11], continuous user engagement [12, 13], user and implementer pluralistic and opposing meanings, where a feature may mean different things, either good or bad to different people [14], and size, location, internal structure, management processes, history, external regulatory environment, culture and leadership [15] have all been listed as factors that influence successful translation of technologies in practice.

In this study we used a participatory evaluation approach, working with key stakeholders in the implementation and maintenance of a new iPad initiative in a large health care organisation. Key users of the iPad initiative were nurses who deliver care in the community. Calzone and colleagues described nurses as the largest global contingent of health providers, and therefore critical stakeholders in translating technology into practice [16].

According to the global literature in implementing such initiatives, having a business plan and a checklist of guiding principles is critical for success of these type of projects [17–19]. Interview with the IT team showed planning of the project followed the Prince 2 project management methodology. Comments from the IT team made clear there was no formal change management strategy in place. There was a time imperative to achieve deployment as quickly as possible, with limited human resources, hence the first come first serve basis approach. The IT team, however, incorporated several critical factors into their planning, namely making users aware of the initiative, training, and following up with updates. While a key lesson is for future implementers to employ an implementation strategy or theory depending on resources and revenues available [5, 17, 20], we also found evidence that informal ‘socialised’ meetings deployed as part of the participatory study contributed greatly to users willingness to sustain adoption. In fact, users prescribed frequent social interactions for future on-going implementation.

Evidence in the literature shows that through mobile technology, community health workers can create value for their organisations; reducing time taken to perform tasks, informing real-time clinical decision-making, removing the need for time consuming journeys, and providing patients with real-time interactive guidance to improve clinical encounters [1, 17, 21–24]. In our study, early adopters were already realising these benefits and wanted to continue the process.

However, there was also evidence of misunderstandings in user and implementer camps, with each misinterpreting what the other might be thinking, which were perceived as challenges. Specifically, we identified three challenges from each of these two perspectives: implementers faced user scepticism and suspicion; clinicians’ misunderstanding and sometimes noncompliance with training processes; and the need to manage user expectations. Users of the iPads sometimes felt overburdened by the work required to use and keep up to date with its functions; they found that the initiative led to some blurring of personal and professional boundaries; and they were dissatisfied with receiving information about the initiative through multiple, dispersed channels.

In their reflections, the IT team recognised that communication was important to the successful ongoing implementation and maintenance of the initiative. This involves communication between implementers and users, and communication between different sections of the implementing team, without which challenges could become even greater. User participants also suggested increased interactivity between the IT team and clinicians, and between themselves, as they discovered through the focus group sessions that they could learn from each other and adapt to the devices by sharing their experiences.

The results therefore suggests a need for the IT team and primary users to engage in a better, more collaborative dialogue to establish common language and co-construct the initiative for long-term purposes. Co-construction as a concept emphasises how value can be created by involving stakeholders in a product or service (artefact) design through collaboration on ideas and feedback. However, rather than viewing the artefact as central to the innovation, co-construction suggests that we view how stakeholders use social relationships (interactivity) to shape the artefact as the innovation, because it is *how* the artefact is shaped that can cause its failure or success [10, 25]. Pinch and Bijker [26] take this concept further by arguing for the need to look at which stakeholders are involved in shaping the artefact, asking, for example, who is included in the collaboration, and how does this evolve into determining whether the artefact has been accepted or rejected?

For co-construction to be effectively applied to the iPad initiative, interactivity needed to exist at many different levels, such as between the different teams in the IT department, between the IT team and users, between users in the same and different directorates, and between different clinical groups and levels. García-Goñi and colleagues found that different groups are usually motivated differently during implementation and adoption depending on their roles [27]. By using a participatory methodology, we have shown that while both the IT team and the users recognise the benefits of the initiative and are keen for its longevity, common misunderstandings can occur between the stakeholder groups which could critically influence how the artefact is shaped, thereby determining its acceptance or rejection by users in the long-term.

Nurses spend more time with patients than any other health care practitioner [1]. As a result, for their easy adaptation to and acceptance of a technology designed to improve working practices it is crucial to consider how they co-construct that technology. To extend and improve on the success of the iPad initiative, improved dialogue and collaboration between stakeholders in informal socialised settings was critical.

### Strengths and limitations

Participatory techniques allowed a deep involvement from stakeholders in the study, with excellent access to user groups, and the opportunity to study a live initiative. However, they can introduce bias in relation to how the study participants were selected and there is a possibility that involving stakeholders in all stages of the study skewed the results in a different direction to another evaluation model. The group nature of eliciting information created the potential that not every participant felt free to express all their experiences, and because this was not longitudinal data from a prospective study we were not able to study changes over time. As this is qualitative study, the small sample restricts findings from being generalised to other implementation and adoption experiences. At the time of undertaking the project the Trust was at the vanguard in terms of iPad deployment methodology. Apple was still developing its software and best practices to support large scale deployments in enterprise organisations. The provider of the Trust’s EHR was also at an embryonic stage in terms of app development / deployment methodologies. As such, many of the technical / support difficulties experienced by the Trust were the result of being at the forefront of introducing transformational solutions. Due to advances made over the past four years these technical / support difficulties are now less likely to be an issue for organisations embarking on a similar project.

## Conclusions

In this study, users identified many benefits which have helped the translation of the new innovation into practice. However, by using the participatory technique to study the implementing team and primary user attitudes, this study has revealed that common misunderstandings can occur between stakeholders when a technological service is being developed through little or no socialised (interactive) engagement. Lack of interactivity between many stakeholder groups can cause people to misinterpret what others might be thinking, which causes misunderstandings and can influence whether the service is ultimately accepted or rejected. We therefore suggest the need for increased and ongoing dialogue between different levels of stakeholder groups in the form of socialised engagements, so as to increase processes involved in co-constructing the iPad initiative for acceptance and sustainability.

## Additional file

Additional file 1: Appendix 1 group sessions’ template. (DOCX 20 kb)

## Data Availability

The datasets used and/or analysed during the current study are available from the corresponding author on reasonable request.

## Abbreviations

CUREC: Central University Research Ethics Committee (CUREC)
EHR: Electronic Health Records
IAPT: Improving Access to Psychological Therapies
IT: Information Technology
NHS: National Health Service
PC-MIS: Patient Case Management Information System
