# Supplementary material for: Harnessing mobile devices to support the delivery of community-based clinical care: a participatory evaluation
Source: BMC Med Inform Decis Mak. 2019 Jul 16;19:134. doi: 10.1186/s12911-019-0869-x (PMC6636105; doi:10.1186/s12911-019-0869-x)
Supplement: Supplementary file 1 — Appendix 1 group sessions’ template. (DOCX 20 kb) [file 12911_2019_869_MOESM1_ESM.docx]

# Additional file 1: Appendix 1 – group sessions’ template

**Pre-discussion**

1. Introduce myself
2. Explain purpose of the study (your EXPERIENCES WITH THE iPad)
3. Idea of focus group, is to have you discussing your experiences among yourself, and I will be in the background and step in from time to time so please feel free to talk among yourselves.
4. Any questions from the Participant Information Sheet
5. Consent form completion
6. Record

**Discussion**

1. Participant’s introduction (Name and position).
2. Before this IPad, did any you have a tablet?
   1. Prompt: do you still use the tablet alongside this one?
3. Operating the iPads.
   1. In general how have you found operating the iPads on a daily basis?
      1. Prompt: Things like turning is on/off, wi-fi, downloading apps, how has been going?
4. Apps
   1. I’m not familiar with the iPad so I’m going to rely on you to first of all tell me functions of the iPad. Could you start my mentioning some of the key App you have been using?
      1. Prompt: FaceTime, Care Notes (EHR), keynote, pages, numbers.
5. How have the Apps influenced how you normally work?
   1. Which apps have you particularly found useful and why?
   2. How about the electronic health records (EHR) app; how is that benefiting your work
6. The iPad in general
   1. How would say the IPads have changed your previous methods of working? Has anyone of you experienced changes in your workload and workflow or changed mobility in how usually operate?
   2. In terms of delivering care in the community, how has the iPad has supported this?
   3. Have you been faced with any challenges?
7. Who here thinks the iPad has helped how and where you access information
8. Did you find that being able to access information anywhere has helped?
9. How about time taken
10. How does it compare with the old ways of doing things
11. What else has the iPad been helpful for?
12. Do you use for your personal things like set up yahoo account and download apps?
13. Has being able to use it for both professional and personal purposes helped at all?
14. Further improvements
    1. Which aspects of the iPad need improving, if any?

**Post-discussion**

1. Voting (so we will end our session by voting)
2. (Raise hands) Who thinks using their iPads has help them their work in general
3. Who thinks the iPad has helped in mobile working?
4. Who has concerns about iPad not helping work?
5. Who thinks being able to access EHR on the iPad has been helpful?
6. Who thinks the apps need improving?
7. Who would recommend it to colleagues?
